# ‘Bingo’—a large language model- and graph neural network-based workflow for the prediction of essential genes from protein data

**DOI:** 10.1093/bib/bbad472

**Published:** 2023-12-27

**Authors:** Jiani Ma, Jiangning Song, Neil D Young, Bill C H Chang, Pasi K Korhonen, Tulio L Campos, Hui Liu, Robin B Gasser

**Affiliations:** Department of Veterinary Biosciences, Melbourne Veterinary School, The University of Melbourne, Parkville, Victoria 3010, Australia; School of Information and Control Engineering, China University of Mining and Technology, Xuzhou 221116, China; Department of Veterinary Biosciences, Melbourne Veterinary School, The University of Melbourne, Parkville, Victoria 3010, Australia; Monash Biomedicine Discovery Institute and Department of Biochemistry and Molecular Biology, Monash University, Melbourne, Victoria 3800, Australia; Department of Veterinary Biosciences, Melbourne Veterinary School, The University of Melbourne, Parkville, Victoria 3010, Australia; Department of Veterinary Biosciences, Melbourne Veterinary School, The University of Melbourne, Parkville, Victoria 3010, Australia; Department of Veterinary Biosciences, Melbourne Veterinary School, The University of Melbourne, Parkville, Victoria 3010, Australia; Department of Veterinary Biosciences, Melbourne Veterinary School, The University of Melbourne, Parkville, Victoria 3010, Australia; Bioinformatics Core Facility, Instituto Aggeu Magalhaes, Fundaçao Oswaldo Cruz (IAM-Fiocruz), Recife, Pernambuco, Brazil; School of Information and Control Engineering, China University of Mining and Technology, Xuzhou 221116, China; Department of Veterinary Biosciences, Melbourne Veterinary School, The University of Melbourne, Parkville, Victoria 3010, Australia

**Keywords:** essential gene prediction, large language model, graph neural network, adversarial training, biological interpretation

## Abstract

The identification and characterization of essential genes are central to our understanding of the core biological functions in eukaryotic organisms, and has important implications for the treatment of diseases caused by, for example, cancers and pathogens. Given the major constraints in testing the functions of genes of many organisms in the laboratory, due to the absence of *in vitro* cultures and/or gene perturbation assays for most metazoan species, there has been a need to develop *in silico* tools for the accurate prediction or inference of essential genes to underpin systems biological investigations. Major advances in machine learning approaches provide unprecedented opportunities to overcome these limitations and accelerate the discovery of essential genes on a genome-wide scale. Here, we developed and evaluated a large language model- and graph neural network (LLM–GNN)-based approach, called ‘Bingo’, to predict essential protein-coding genes in the metazoan model organisms *Caenorhabditis elegans* and *Drosophila melanogaster* as well as in *Mus musculus* and *Homo sapiens* (a HepG2 cell line) by integrating LLM and GNNs with adversarial training. Bingo predicts essential genes under two ‘zero-shot’ scenarios with transfer learning, showing promise to compensate for a lack of high-quality genomic and proteomic data for non-model organisms. In addition, the attention mechanisms and GNNExplainer were employed to manifest the functional sites and structural domain with most contribution to essentiality. In conclusion, Bingo provides the prospect of being able to accurately infer the essential genes of little- or under-studied organisms of interest, and provides a biological explanation for gene essentiality.

## INTRODUCTION

Essential genes are those that are crucial for life in organisms and cells [1]. These genes are usually involved in relative conserved biological processes and pathways [2–4]. Thus, understanding their functional roles has important implications for understanding the minimum core set of genes (‘essentialome’) in eukaryotic organisms [5, 6] and can enable the discovery of interventions (e.g. [6–9]).

The multicellular model organisms *Caenorhabditis elegans* and *Drosophila melanogaster* have been used for decades to study fundamental biological processes and principles for multicellular (metazoan) organisms employing functional genomic (i.e. gene knock-down and knock-out) tools [8, 9]. Underpinned by the knowledge of their complete genomes, these two species have acted as surrogates to experimentally infer and/or study essential genes. Most eukaryotic organisms, including most parasites, are not amenable to functional genomics, because they cannot be cultured continuously, or readily manipulated genetically, such that the identification of essential genes is usually not possible using conventional laboratory methods. Therefore, devising reliable computational methods to identify and classify essential genes in non-model organisms has major fundamental and applied implications, particularly for the identification drug and/or vaccine targets in eukaryotic parasites [10].

Various research teams have made advances in the area of bioinformatics [11–15], but the computational inference of essential genes remains a challenge due to its ‘context-dependency’ [10, 16] and a limited understanding of which gene features relate to gene essentiality [17]. Nonetheless, some researchers have indicated that machine-learning (ML) methods could significantly advance this area [18–20], and some of our own studies have also shown promise in predicting and prioritizing essential genes in *C. elegans* and/or *D. melanogaster* using ML models, including eXtreme Gradient Boosting (XGB), gradient boosting machines (GBM) and random forest [21–24]. This work overcame previous limitations relating to annotations based on experimental (phenomic) datasets, the discovery of predictors from large-scale omics datasets, and parameter tuning and cross validation, and provided a basis for the prediction of essential genes particularly in ecdysozoans—to which both *C. elegans* and *D. melanogaster* belong [10].

The use of deep-learning (DL) algorithms is highly likely to further enhance accuracy of the computational prediction, identification and/or prioritization of essential genes. Deep neural networks, such as recurrent neural networks (RNNs), contextual embedding-based convolutional neural networks (CNNs) and topological feature-coupled graph neural networks (GNNs), have the potential to automatically extract informative (semantic) features from diverse datasets, including protein sequences, protein–protein interactions (PPIs) and biological information or data. The architectures of these networks can enable the discover of cryptic patterns that are linked to gene essentiality. For example, extending a previous study [25], Zeng *et al*. [15] used a DL framework to identify essential proteins by integrating three types of biological information. Specifically, they used the node2vec framework for extracting topological features from PPIs; applied the RNN-based bidirectional long short-term memory (BiLSTM) to capture contextual information from gene expression data and employed high-dimensional indicator vector to characterize subcellular localization. Recently, Schapke *et al*. [26] proposed Essential Prediction Graph Attention Network (EPGAT), an attention-based GNNs approach for essentiality prediction based on graph attention networks (GATs) employing graph-structured data. EPGAT directly learns gene essentiality patterns from PPI networks, and integrates other evidence from multi-omics data encoded as node attributes. EPGAT was shown to outperform network-based and shallow ML-based approaches.

In spite of the promise of these approaches, some challenges remain for the DL-based prediction of essential genes, including: (i) Most techniques rely heavily on manually engineered features, orthology and/or biological networks, but extensive datasets are often scant for most organisms, and novel genes encoding ‘orphan proteins’ [27, 28] are common; (ii) PPI datasets (from different studies) are produced using a variety of methods, and are often not directly comparable. Models based on PPI cannot extend themselves to genes that does not exist in current PPI network; (iii) Existing models for the prediction of essential genes lack interpretability [29], likely leading to a reduction in transparency and understanding of the models and predictive results.

In the present study, we tackled these problems by establishing a DL workflow with simple input, rich embeddings, accurate prediction—which allows the model to be explained. So, we focused on methods that take protein sequence as inputs and did not focus on, or compare to those rely on protein networks. Under this paradigm, we designed and evaluated the performance of a large language model- and graph neural network (LLM–GNN)-based workflow—called ‘Bingo’—for the prediction of essential genes exclusively from their respective protein sequences in the metazoan model organisms *C. elegans* and *D.*  *melanogaster* as well as for *Mus musculus* and *Homo sapiens* (a HepG2 cell line). We employed a pre-trained protein language model—Evolutionary Scale Modeling-2 (ESM-2) [30], GNNs and a classification module, with adversarial training, to predict essential protein-encoding genes. Then, we utilized the attention mechanisms of ESM-2 and GNNExplainer [31] to explore the link between protein motifs (both sequential and structural) and gene essentiality. This approach provides the prospect of being able to accurately infer the essential genes of little- or under-studied organisms of interest, and offers a biological explanation for gene essentiality.

## MATERIALS AND METHODS

### Datasets

Here, we selected data representing four eukaryotic species, including *C. elegans*, *D. melanogaster*, *M. musculus* and *H. sapiens* in our study for model training and performance evaluation. *Caenorhabditis elegans* and *D. melanogaster* are well characterized model organisms in relation to essential genes [10, 11, 17]. As a mammalian model organism, *M. musculus* is somewhat more complex biologically and shares significant genetic and physiological similarities to humans, having the potential of shedding light on studying fundamental biological processes shared by mouse and human [32]. In addition to these model organisms, we also incorporated the HepG2 cell line into our study. HepG2, a widely recognized human hepatocellular carcinoma cell line, serves as a prominent model for liver research [33]. Highly characterized and established for *in vitro* experiments, it can offer relevant insights into liver biology and pathways.

**Figure 1:**
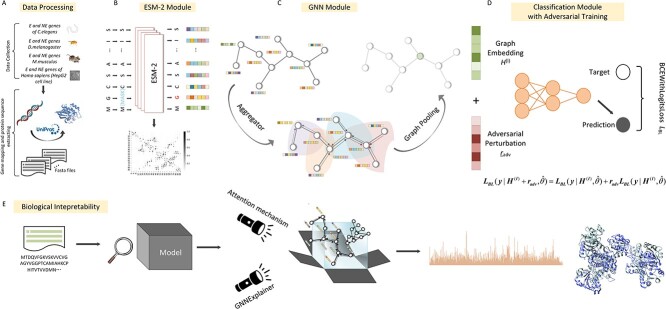
Schematic representation of the Bingo workflow developed here for the prediction of essential protein-coding genes. (**A**) Data processing. We extracted protein-coding essential genes and non-essential genes of *C. elegans*, *D. melanogaster*, *M. musculus* and the *H. sapiens* HepG2 cell line from the OGEE database. We undertook one-to-one mapping of the Ensembl gene identifier to the protein UniProt identifier, and eliminated low-quality or uncurated protein sequences. For high-quality protein sequences, we retrieved FASTA files. (**B**) ESM-2 module. Using these FASTA files as input, ESM-2 learns dependencies between the residues by solving the task of filling in randomly masked amino acids, generating residue-level feature matrix and protein contact map for downstream analysis. (**C**) GNN modules. Embracing residue-level features as node features, contact map as edges, GNNs propagate the residue-level features in a message passing and aggregation manner. As a result, GNNs generate a comprehensive graph embedding that compress both semantically enriched and structurally grounded information. (**D**) Classification module and adversarial training. Final prediction using Multilayer Perceptron (MLP) layers with cross-entropy loss was applied to optimize the model by training. To enhance the robustness of the workflow, and ensure stable prediction results, adversarial training was applied to individual GNNs by adding adversarial perturbation to embeddings, where the worst-case perturbation is determined by solving the min–max objective function. (**E**) Biological interpretability module. The attention mechanism and GNNExplainer was used to explore the link between gene essentiality and key functional or structural domains, motifs and/or sites in proteins.

We designed a data processing pipeline (Figure 1A) for retrieving their protein sequences. First, we extracted protein-coding genes of *C. elegans*, *D.*  *melanogaster*, *M. musculus* or *H. sapiens* (i.e. the HepG2 cell line; ATCC HB-8065) from the online gene essentiality *database* (OGEE) [34] which are known to be essential or non-essential based on published evidence from experimental studies. Second, we generated a gene card for each gene and mapped gene Ensembl identifiers to UniProt identifiers using the software MyGene [35]. Third, for ensuring one-to-one mapping from genes to protein sequences, we selected the isoform with the highest annotation score. The processing scripts are available at https://github.com/jianiM/Bingo.

### The ‘Bingo’ methodology

Here, we designed and critically assessed the Bingo framework (Figure 1B–E) for the prediction of gene essentiality exclusively from protein sequence data. Bingo takes protein sequences as input and employs a pre-trained language model ESM-2, GNNs and classification module. ESM-2 learns dependencies among amino acid residues of proteins by solving the task of filling in randomly masked amino acids, generating residue-level feature matrix and a protein contact map (Figure 1B). Using residue-level information as node features, contact map as edges, GNNs propagate the residue-level features (in a message passing and aggregation manner), generating comprehensive graph embedding that compresses both semantically enriched and structure information (Figure 1C). A fully connected, linear network, with cross-entropy loss, was applied to optimize the model during training. Adversarial perturbation was also applied to GNN embeddings to establish the ‘worst-case’ perturbation by solving the min–max objective function (Figure 1D). Subsequently, the attention mechanism was used to relate the (normalized) importance of an amino acid residue in a protein sequence to gene essentiality, and GNNExplainer [31] was employed to infer which structural domains/sites in a protein link most to this essentiality (Figure 1E). The key components of Bingo are described in the following:

#### ESM-2

Here, we used the pre-trained protein language model ESM-2 [30] to ‘understand’ functional and evolutionary information embedded in protein sequences, and captured dependencies among the amino acids to generate residue-level features and a contact map. Specifically, ESM-2 was trained on >180 million proteins in the UniRef database at scales from 8 million parameters up to 15 million parameters by solving the task of filling in randomly masked amino acids in protein sequences. For a set of training protein sequences *S*, ESM-2 maximizes the following masked language modelling (MLM) objective:


(1) \begin{equation*} {L}_{MLM}\left(S,\Theta \right)=\underset{s\sim S}{E}\underset{mask}{E}\sum \limits_{i\in mask}\log P\left({s}_i|{s}_{j\notin mask};\Theta \right) \end{equation*}

where *s* = (*s*_1_, *s*_2_,…, *s_L_*) is the protein sequence with *L* residues, mask is a randomly generated mask that includes 15% missing amino acids of positions *i* in sequence *s*. The model tries to identify missing residues *s*_i_ from the context ${s}_{j\notin mask}$ .Θ is the model parameters. By predicting the missing tokens of the corrupted sequence, ESM-2 must identify the dependencies between the masked site and unmasked site. By leveraging the well pre-trained ESM-2 model on essential/non-essential gene-encoded proteins, Bingo generates residue-level feature matrix $X\in{\mathbb{R}}^{L\times c}$ where *c* denotes the dimension of residue features and protein contact map $A\in{\mathbb{R}}^{L\times L}$ for sequence *s*. Both are essential inputs for GNN, which is described below.

#### GNN

For the present workflow, we assessed four different GNN models, namely graph convolutional network (GCN) [36], GAT [37], graph sample and aggregated network (GraphSAGE) [38] and graph isomorphism network (GIN) [39] to propagate residue-level features among amino acids, which are structurally proximal to one another. The protein contact map captures the spatial proximity between every conceivable pair of amino acids within each protein. This compact two-dimensional, symmetrical matrix is derived from the three-dimensional interactions among amino acids in the protein’s structure [40]. To cast the contact map into a graph, we set a specific threshold (γ) to ensure that 20% of the residue pairs can be retained in the contact map matrix. For example, for two residues *s*_i_ and *s*_j_, if | *s*_i_ – *s*_j_ | < γ, then *A_ij_* = 1, (*i*, *j* = 1,2,…,*L*) and *A_ij_* = 0, vice versa. Considering that some residues that are distant from one another in primary sequence but are close in structure, we utilized GNNs to further extract the structure-derived embeddings that are complementary to the primary features. Taking the contact map $A\in{\mathbb{R}}^{L\times L}$ and residue-based feature matrix $X\in{\mathbb{R}}^{L\times c}$ as inputs, GNNs can learn the protein-specific encoder to extract structural features and output the probability of a genes’ essentiality. The detailed description of the four GNN models and how they “were” implemented are given in the Supplementary Material, available online at http://bib.oxfordjournals.org/.

#### Adversarial training

Adversarial training [41] is a regularization method for the neural networks to learn to increase the robustness of adversarial attacks. Bingo extends the adversarial training strategy to GNN models to prevent overfitting. Of the proposed adversarial training methods available for image and text embedding, we leveraged the fast gradient method (FGM) [42]. As shown by the Equation (2), the BCEWithLogitsLoss function *L_BL_* is used to train our model as a base loss function: 


(2) \begin{equation*} {L}_{BE}\left(y|s,\theta \right)=-y\log \left(\sigma \left(p\left(s;\theta \right)\right)\right)-\left(1-y\right)\log \left(1-\sigma \left(p\left(s;\theta \right)\right)\right) \end{equation*}

where (*s*, *y*) denotes a training sample; *s* is the input training sequence; *y* is the true label; *θ* denotes the model parameters; $p\left(s;\theta \right)$ denotes the prediction probability and *σ* is the sigmoid function. For basic training, the optimal model parameter can be obtained through the backpropagation procedure: 


(3) \begin{equation*} {\theta}^{\ast }=\arg \underset{\theta }{\min }\ {E}_{\left(s,y\right)\sim D}L\left(y|s,\theta \right) \end{equation*}

where *D* is the training set.

In the previous work, the adversarial perturbation was directly applied to the text embeddings of sequence data [43, 44]. The learning procedure for finding the optimal adversarial perturbation ${r}_{adv}^{\ast }$ and model parameters ${\theta}^{\ast }$ is commonly formulated as a min–max optimization problem as follows: 


(4) \begin{equation*} {\theta}^{\ast },{r}_{adv}^{\ast }=\underset{\theta }{\min }\ {E}_{\left(s,y\right)\sim D}\left[\underset{r_{adv}}{\max }{L}_{BL}\left(y|f(s)+{r}_{adv},\theta \right)\right] \end{equation*}

where we used *f* (*s*) to represent the embedding feature of sequence *s*, and *r_adv_* denotes adversarial perturbation.

In Bingo, we applied the adversarial perturbation to the GNN layers, and solved the following objective function: 


(5) \begin{equation*} {\theta}^{\ast },{r}_{adv}^{\ast }=\underset{\theta }{\min }\ {E}_{l=1,2,...,L}\left[\underset{r_{adv}}{\max }{L}_{BL}\left(y|{H}^{(l)}+{r}_{adv},\theta \right)\right] \end{equation*}

Following a two-stage process at each training step to obtain the worst-case perturbation ${r}_{adv}^{\ast }$ and optimal model parameter θ^*^, we initially obtained ${r}_{adv}^{\ast }$ by solving: 


(6) \begin{equation*} \underset{r_{adv}}{\max }\ {L}_{BL}\left(y|{H}^{(l)}+{r}_{adv},\hat{\theta}\right),l=1,2,...,L \end{equation*}

where $\hat{\theta}$ is a temporarily fixed value of *θ*.

However, the exact maximization with respect to *r_adv_* is intractable for GNNs. Thus, for FGM, we linearized ${L}_{BL}\big(y|{H}^{(l)}+{r}_{adv},\hat{\theta}\big)$ around *H*^(*l*)^ with an L_2_-norm constraint, that is:

${L}_{BL}\big(y|{H}^{(l)}+{r}_{adv},\hat{\theta}\big)={L}_{BL}\big(y|{H}^{(l)},\hat{\theta}\big)+{r}_{adv}{L}_{BL}\big(y|{H}^{(l)},\hat{\theta}\big)$


(7) \begin{equation*} \text{where}\ {\left\Vert{r}_{adv}\right\Vert}_2\le \varepsilon \end{equation*}

The adversarial perturbation causes the loss function to grow by ${r}_{adv}{L}_{BL}\big(y|{H}^{(l)},\hat{\theta}\big)$. Maximizing ${L}_{BL}\big(y|{H}^{(l)}+{r}_{adv},\hat{\theta}\big)$ is equivalent to maximizing ${r}_{adv}{L}_{BL}\big(y|{H}^{(l)},\hat{\theta}\big)$. Thus, by considering the L_2_-norm constraint on *r_adv_*, we assigned: 


(8) \begin{equation*} {r}_{adv}^{\ast }=\varepsilon \frac{\nabla_{H^{(l)}}{L}_{BL}\left(y|{H}^{(l)},\hat{\theta}\right)}{{\left\Vert{\nabla}_{H^{(l)}}{L}_{BL}\left(y|{H}^{(l)},\hat{\theta}\right)\right\Vert}_2} \end{equation*}

After obtaining the optimal perturbation ${r}_{adv}^{\ast }$, ${\theta}^{\ast }$ can be achieved by solving:


(9) \begin{equation*} \underset{\theta }{\min }{E}_{l=1,2,...,L}{L}_{BL}\left(y|{H}^{(l)}+{r}_{adv}^{\ast },\theta \right)\Big] \end{equation*}

## RESULTS

Here, we curated the gene and protein datasets for *C. elegans*, *D. melanogaster*, *M. musculus* and HepG2 cells; compared and intuitively analyzed the performance of Bingo with Transformer [45], BiLSTM [46] and CNN [47], using separately balanced and imbalanced datasets, employing a 10-fold cross validation; assessed the performance of Bingo for ‘zero-shot’ (i.e. *de novo*) cross-species prediction and cross-domain prediction for *C. elegans*; evaluated and analyzed the contribution of each module in our model; elucidated how each module worked and provided biological insights in explaining our model’s decision. All models in this section were trained on an NVIDIA A100 with 80 GB of memory; early stops were controlled by validation loss.

### Curated datasets obtained

First, we extracted data from OGEE and identified protein sequences linked to essential and non-essential genes (Table 1).

**Table 1:** Statistical summary of essential and non-essential protein-coding genes from *C. elegans*, *D. melanogaster*, *M. musculus* and *H. sapiens* (HepG2 cell line)

Dataset representing organism/cell line	No. of essential genes	No. of non-essential genes
*C. elegans*	578	13 104
*D. melanogaster*	365	7178
*M. musculus*	2016	7491
*H. sapiens (HepG2* cells; ATCC HB-8065)	877	16 040

We primarily employed balanced datasets, while also taking imbalanced datasets into account when evaluating performance. Specifically, we obtained balanced datasets for individual species by randomly sampling non-essential genes; this sampling set was the same in size as the set representing essential genes.

### Predictive performance of Bingo using balanced and imbalanced datasets

**Figure 2:**
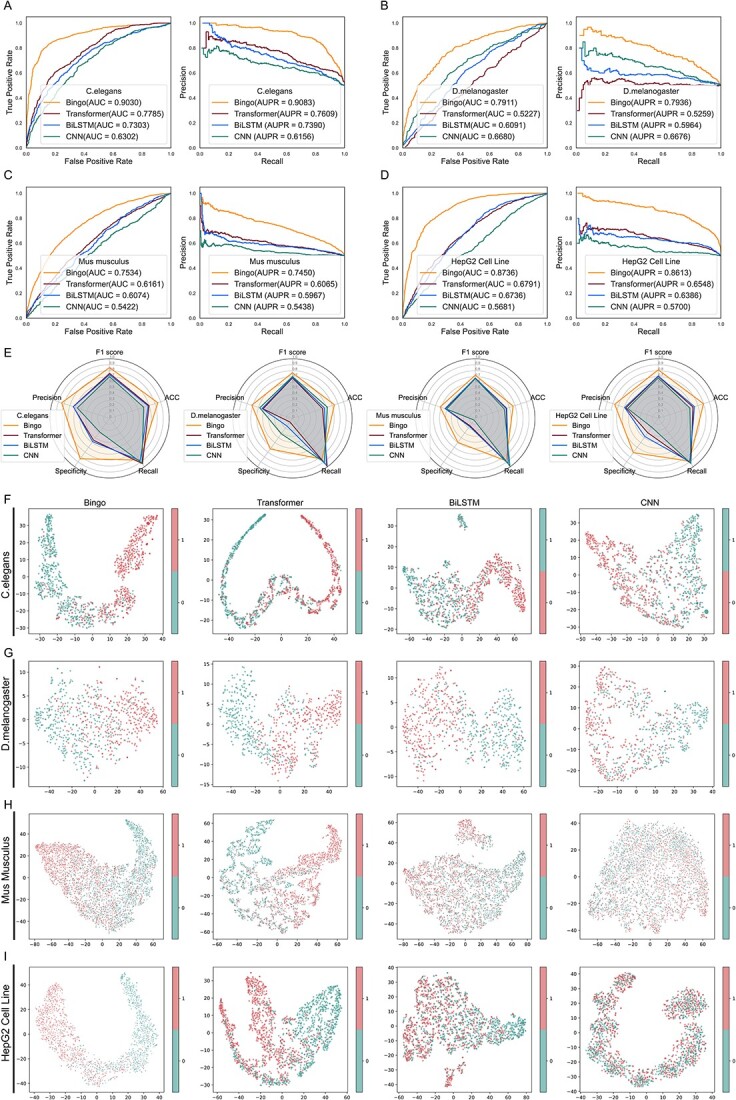
Comparison of the overall performance of Bingo and other methods using balanced data (10-fold cross validation). (**A–D**) The ROC and PR curves of Bingo (and its competing methods on balanced datasets for *C. elegans*, *D. melanogaster, M. musculus* and the *H. sapiens* HepG2 cell line. (**E**) Performances of Bingo, Transformer, BiLSTM and CNN using four balanced datasets, in terms of ACC, Precision, Recall, F1 score and Specificity (10-fold cross validation). (**F–I**). Feature distributions for Bingo, Transformer, BiLSTM and CNN using balanced datasets for *C. elegans*, *D. melanogaster*, *M. musculus* and HepG2 cells, respectively. Red and green dots denote essential (positive) and non-essential (negative) genes, respectively.

We compared the performance of Bingo with those of Transformer, BiLSTM and CNN using balanced datasets (representing *C. elegans*, *D. melanogaster*, *M. musculus* and the human HepG2 cell line) on a 10-fold cross-validation test. The rationale for using Transformer, BiLSTM and CNN as comparative benchmark models is given in the Supplementary Material, available online at http://bib.oxfordjournals.org/. The models were trained and evaluated using the same training sets and test sets. The performance was mainly measured using Area Under the Receiver Operating Characteristic Curve (AUC) and Area Under the Precision-Recall Curve (AUPR), followed by other measurements such as F1 score, Accuracy (ACC), Recall, Specificity and Precision. How to calculate them can be found in Supplementary File, available online at http://bib.oxfordjournals.org/. Figure 2A–E illustrates the overall comparative performances between Bingo and each of the three other methods using balanced protein datasets for *C. elegans*, *D. melanogaster*, *M. musculus* and the HepG2 cell line.

As Figure 2A–D shows, Bingo achieved the best AUC and AUPR values, leading by a 12.5–27.3% for AUC, 14.7–29.3% for AUPR for *C. elegans*, 12.3–26.8% for AUC, 12.6–26.8% for AUPR for *D. melanogaster*, 13.7–21.1% for AUC, 13.9–20.1% for AUPR for *M. musculus*, 19.45–30.45% in AUC and 20.65–29.13% in AUPR for the HepG2 cell line, respectively. Figure 2E further demonstrates the superiority of Bingo over Transformer, BiLSTM and CNN, in terms of the F1 score, Precision, Recall, ACC and Specificity for all four balanced datasets. Taken together, these results demonstrated superior performance of Bingo compared with Transformer, BiLSTM and CNN. 

To explain why Bingo performed better than these other methods, we assessed the distribution of the feature embedding space for Bingo as well as for Transformer, BiLSTM and CNN for all test sets generated by stratified 10-fold cross validation, revealing the models’ capabilities to exploit the underlying pattern of feature space and the discriminability to differentiate essential from non-essential genes. Figure 2F–I shows the t-distributed stochastic neighbour embedding (t-SNE) visualization [48] results of them on *C. elegans*, *D. melanogaster*, *M. musculus* and HepG2 cells, respectively, in which red dots represent the positive samples (essential genes), while green dots represent the negative samples (non-essential genes). As can be seen in Figure 2F and I, the feature space of essential genes and that of non-essential genes are distinct. In addition, the samples within one cluster are compact rather than disperse. In contrast, the samples of feature space generated by Transformer are somewhat mixed, whereas for BiLSTM and CNN, they are connected and totally mixed, preventing clear classification. As for Figure 2G and H, although the feature spaces for Bingo are not as discriminative as those achieved using *C. elegans* or HepG2 cell data, they are clearer than those generated by the three other methods using data from *D. melanogaster* and *M. musculus*. Moreover, the t-SNE performance aligned perfectly with the comparative results shown in Figure 2A–E. Thus, the performance of Bingo can be attributed to its excellent ability to recognize and harness the distinctive feature patterns of essential and non-essential genes in distinct species. The predictive performance and analysis of four imbalanced datasets (i.e. for *C. elegans*, *D. melanogaster*, *M. musculus* and HepG2 cells) using a stratified 10-fold cross validation are present in the Supplementary Material, available online at http://bib.oxfordjournals.org/. Figure S1 shows that our workflow achieved the best performance for the prediction of essential genes compared with the three other methods using imbalanced datasets.

### Assessing the performance of Bingo for ‘zero-shot’ (i.e. *de novo*) prediction of essential genes

**Figure 3:**
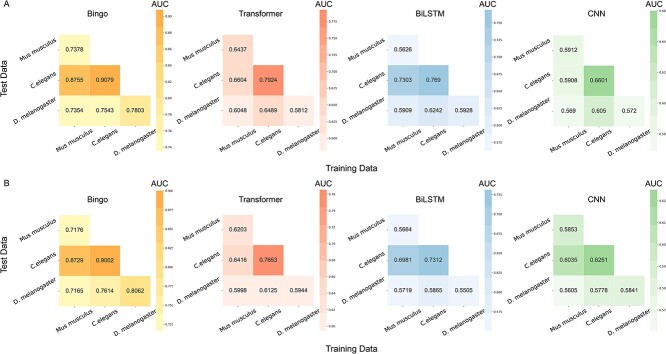
AUC and AUPR values for ‘zero-shot’ predictions of essential genes. (**A**) AUC heatmap of cross-species transfer learning with Bingo, Transformer, BiLSTM and CNN. (**B**) AUPR heatmap of cross-species transfer learning using Bingo, Transformer, BiLSTM and CNN.

Here, we evaluated Bingo to cross-predict essentiality from one species to another using transfer learning on their balanced datasets. Specifically, in the model training process, we divided the large-scale samples into the training dataset and validation dataset with 8:2 ratio. By temporarily hiding the essential gene annotations of their samples, the small-scaled organism was regarded as ‘unseen’ one. Applying the well-trained model into ‘unseen’ species, we obtained the ‘zero-shot’ prediction results. For each model, we used AUC and AUPR to measure the overall performance for cross-species transfer learning experiments (Figure 3).

As a result, Bingo achieved higher AUC and AUPR scores than did other models for transfer learning for all metrics (Figure 3), highlighting its applicability and adaptability in ‘zero-shot’ prediction and its potential to provide reliable essential gene predictions for ‘unseen’ species. In addition, for each model, the performance for the ‘*C. elegans*–*D. melanogaster*’ pair (i.e. *C. elegans* is the source dataset and *D. melanogaster* is the target dataset) consistently surpassed that achieved for the ‘*M. musculus*–*D. melanogaster*’ pair. This finding indicates that *C. elegans* data are a more reliable source to train quality models to learn and prioritize essential genes in an ‘unseen’ species.

Cross-species prediction was conducted using the *C. elegans* data as a training set for the prediction and prioritization of essential genes. We compared Bingo with three protein sequence-derived state-of-the-art (SOTA) methods: DeepCellEss [49], Essential Protein-Ensembl Deep Learning (EP-EDL) [50] and Essential Protein-Gradient Boosting Decision Tree (EP-GBDT) [51], using a ‘zero-shot’ cross-domain prediction for *C. elegans*. The rationale for selecting DeepCellEss, EP-EDL and EP-GBDT as competitive SOTA methods is given in the Supplementary File, available online at http://bib.oxfordjournals.org/. In addition, a Top-K gene strategy was used as an evaluation criterion to intuitively assess the predictive capability of cross-domain predictions using *C. elegans* data. Detailed information regarding the Top-K gene scheme and how Bingo, DeepCellEss, EP-EDL and EP-GBDT were implemented is given in the Supplementary File, available online at http://bib.oxfordjournals.org/. Figure S2 compares the results achieved using the Top-K gene scheme.

### Both ESM-2 and GAT improved prediction performance

**Figure 4:**
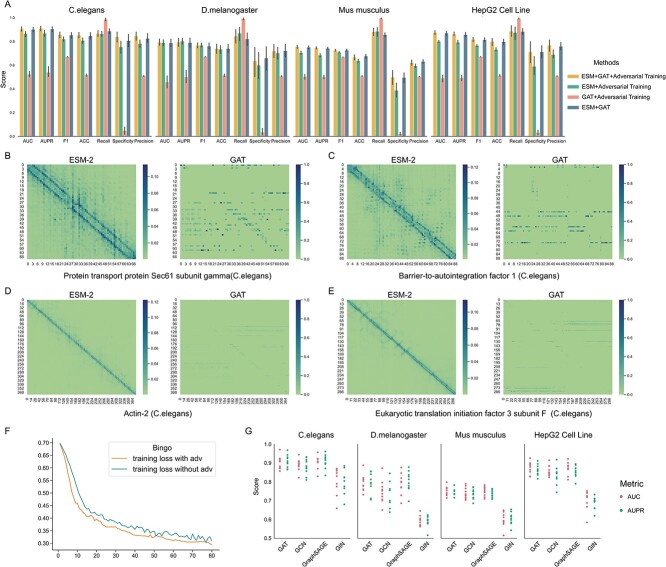
Analysis of the performance and interpretability of each module of Bingo. (**A**) Performance comparison of Bingo, ‘ESM-2 + Adversarial Training’, ‘GAT + Adversarial training’ and ‘ESM-2 + Adversarial Training’. (**B–E**) Attention maps to illustrate the features captured by ESM-2 and GAT of protein transport protein Sec61 subunit gamma, barrier-to-autointegration factor 1, actin-2 and eukaryotic translation initiation factor 3 subunit F. (**F**) Performance comparison of Bingo employing GAT, GCN, GraphSAGE or GIN for the analysis of datasets for *C. elegans*, *D. melanogaster, M. musculus* and the *H. sapiens* HepG2 cell line. Training curves for Bingo with and without the adversarial training on the *C. elegans* data set. (**G**) 10-fold cross validation of Bingo with GAT, GCN, GraphSAGE and GIN, respectively, for *C. elegans*, *D. melanogaster*, *M. musculus* and the HepG2 cell line.

Here, we wanted to understand how its components contribute to the high performance and adaptability of Bingo. To evaluate the effectiveness of ESM-2, we replaced ESM-2 with simple one-hot encoding scheme, followed by GNN and adversarial training. In addition, to assess the impact of GAT, we systematically deactivated its functionality and employed a linear neural network for the fine-tuning of ESM-2. Throughout the training process, we consistently employed an adversarial training scheme. Figure 4A shows the AUC and AUPR scores of ablation experiments using balanced datasets representing *C. elegans*, *D. melanogaster*, *M. musculus* and HepG2 cells.

As shown in Figure 4A, the ablation studies were conducted to assess the performance of individual module. Comparing these models to Bingo, the ones that involve ESM + adversarial training, GAT + adversarial training and ESM + GAT exhibit varying degrees of performance decline across metrics, except for Recall on *C. elegans*, *D. melanogaster*, *M. musculus* and the HepG2 cell line. Notably, the models without ESM-2 displayed the most significant performance decreases, highlighting the substantial contribution that ESM-2 makes to enhancing the Bingo’s prediction performance. Similarly, GAT also plays a crucial and indispensable role in improving the predictive performance.

### ESM-2 and GAT capture complementary information

Here, we focused on elucidating the reason(s) for its high predictive performance of Bingo. We selected, at random, four protein-coding genes from *C. elegans* and extracted the attention maps of ESM and GAT, and deciphered the contextual or structural independence that they learned from the protein sequence. Figure 4B–E presents the attention maps of protein transport protein Sec61 subunit gamma (UniProt ID: Q19967; Ensembl ID: WBGene00001303), barrier-to-autointegration factor 1 (UniProt ID: Q03565; Ensembl ID: WBGene00000235), actin-2 (UniProt ID: P10984; Ensembl ID: WBGene00000064) and eukaryotic translation initiation factor 3 subunit F (UniProt ID: Q18967; Ensembl ID: WBGene00001229), with each subfigure delineating the attention maps generated by ESM and GAT.

Notably, each entry in the attention map reflects the degree of relevance between two residues along the protein sequence. Intensified colours signify heightened correlation, signifying the importance of the association. In the ESM attention maps, we observed that highly correlated elements are concentrated around the diagonal, indicating that ESM can capture the contextual information of residues by considering their upstream and downstream amino acids within the protein sequence. In contrast, the attention maps generated by GAT exhibited a more dispersed distribution of highly correlated elements. This indicates that GAT can capture the spatial correlations among residues that may be distantly positioned within a protein sequence, but are in close spatial proximity. Remarkably, the contextual correlation information extracted by ESM and spatial proximity information extracted by GAT are complementary, providing a holistic and clear feature space and indicating that ESM and GAT play key roles in enhancing predictive performance.

### Adversarial training shows the robustness of the Bingo workflow

Adversarial training is another key component of Bingo. To investigate its contribution to the model’s performance, we first trained our workflow without adversarial training (see dark blue bar in Figure 4A), and compared its performance with that of the original model. Compared with the performance of the original model, denoted as the orange bars, we found that its contribution to prediction performance improvement is limited. Subsequently, using the training of *C. elegans* data as an example, we further analyzed the loss curves with and without adversarial training (Figure 4F). In Figure 4F, we see that the model with adversarial training achieved lower and a more stable training-loss than that without adversarial training. This indicates that adversarial training promotes a better convergence of the model and mitigates potential overfitting risks, likely eventually enhancing the robustness of our workflow.

### GAT performs best of a range of GNN models

To examine the power of the GNN models GAT, GCN, GraphSAGE and GIN, we ran our workflow with these GNN variants using balanced *C. elegans*, *D. melanogaster*. *Mus musculus* and HepG2 cells datasets on 10-fold cross validation. Figure 4G shows the performance (measured as AUC and AUPR) for each fold using these four balanced datasets. Notably, the performance of *Bingo* with these four GNN variants is consistent with that using the four distinct datasets for both metrics. GAT achieved the best performance with the highest mean AUC/AUPR and the least variation, followed with GraphSAGE, and GCN. GIN did not perform well, which may be attributable to its feature/label invariant for isomorphic graph (limited sensitivity to node-embedding features and high reliance on structure). Specifically, GIN was designed with the concept of graph isomorphism, i.e. for proteins with similar structures (contact map here), GIN generates a very similar graph representation, rendering it unable to differentiate reliably essential from non-essential genes based on protein data alone. On the other hand, GAT, GIN and GraphSAGE are more sensitive to node-embedding and are not totally reliant on structures. They use diverse means of propagating the embedding of nodes – GCN aggregates embedding in a simple way, i.e. averaging the embedding of all neighbours’ features without selection. For each node, GraphSAGE first samples its neighbours and then updates the embedding by concatenating current embedding and aggregated neighbour information. Averaging may cause ‘noise’, while randomly sampled node’s neighbourhoods should mitigate this situation. For this reason, GraphSAGE achieved a better performance than GCN. Compared with GCN and GraphSAGE, it seems that GAT leverages a ‘smarter’ and more reasonable node feature aggregation approach. GAT introduces the attention mechanism during the message passing procedure, assigning weights to node’s neighbours according to their contribution to the control and/or target node. Taken together, these findings indicate that the full exploration of nodes’ embedding underpins the superior performance of our workflow employing GAT.

### Linking protein motifs, domains and sites to essentiality

Many proteins carry out their biological functions through important residues or motifs (e.g. active sites on enzymes, DNA binding sites on transcription factor proteins, binding sites and post-transcriptional modification sites) and functional domains. These elements might relate to the essentiality of a gene. To better interpret the decision-making of a model at the molecular-level, Bingo outputs the normalized importance of an amino acid residue through the attention mechanism of ESM-2, and localizes substructure contributing most via GNNExplainer [31] without residue-level annotation. To gain insights into how Bingo makes predictions at the sequence-level, we first extracted the attention matrix generated by ESM-2, which records the correlations among all residues. Then, we quantified the contribution of each residue by summing up its correlations with all other residues, and submitted the summed value via min–max scaling to derive a normalized value. A higher normalized value indicates a stronger correlation/dependency with the upstream and downstream regions in the protein, thus demonstrating the important role that each residue plays in the determination process. Furthermore, to offer insight into how Bingo makes decision at the structure-level, we applied GNNExplainer to the contact map by detecting the compact connected subgraph with the maximum connectivity.

**Figure 5:**
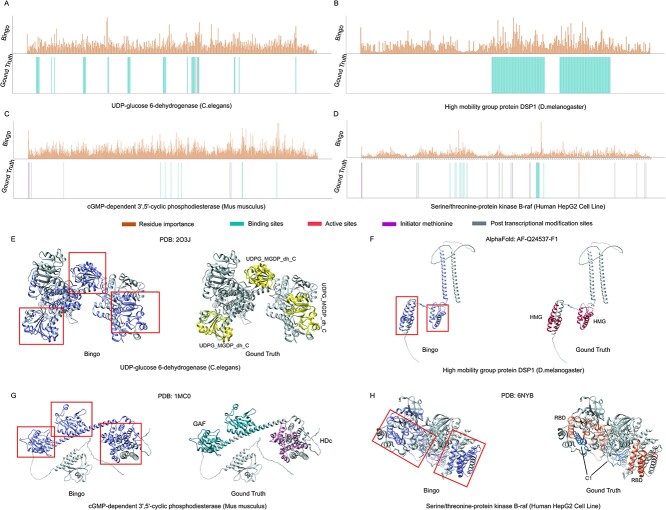
Exploring links between essential genes and their protein features (examples). (**A–D**) Normalized residue importance, generated by attention mechanism and ground truth annotations for UDP-glucose 6-dehydrogenase, high mobility group protein DSP1, cGMP-dependent 3′,5′-cyclic phosphodiesterase and serine/threonine-protein kinase B-raf, respectively. (**E–H**) Most contributed connected domain analyzed by GNNExplainer and ground truth annotations of UDP-glucose 6-dehydrogenase, high mobility group protein DSP1, cGMP-dependent 3′,5′-cyclic phosphodiesterase and serine/threonine-protein kinase B-raf, respectively.

Here, we present examples of essential genes encoding UDP-glucose 6-dehydrogenase (UniProt ID: Q19905; Ensembl ID: WBGene00005022), high mobility group protein DSP1 (Q24537; FBgn0278608), cyclic guanosine monophosphate (cGMP)-dependent 3′,5′-cyclic phosphodiesterase (Q922S4; ENSMUSG00000110195) and serine/threonine-protein kinase B-raf (P15056; ENSG00000157764) from *C. elegans*, *D. melanogaster*, *M. musculus* and HepG2 cells, respectively. Figure 5A–D shows the distribution of the importance scores for individual residues and functional motifs/domains in the protein sequences encoded by these four genes. Figure 5E–H shows the connected subgraphs that contribute most (i.e. with highest importance residue score) for each protein structure analyzed by GNNExplainer, along with the ground truth structural functional domains annotated using SMART Domain tool [52] and the UCSF Chimera tool [53].

UDP-glucose 6-dehydrogenase is an enzyme involved in carbohydrate metabolism for *C. elegans*, utilizing NAD^+^ as a co-factor for oxidizing UDP-glucose to UDP-glucuronic acid [54]. Its activity and interactions with other proteins are primarily influenced by NAD^+^ binding sites, catalytic sites, and the UDP-binding domain. Figure 5A and E shows that positions with higher residue importance align well with NAD^+^ binding sites (green) and catalytic sites (red) at the sequence level, while the UDP functional domain can also be found in the most-contributed connected subgraph of its whole structure. In addition, the high mobility group protein DSP1 functions as a transcriptional co-activator of *D. melanogaster*, which is characterized by its ability to bind to DNA and regulate gene expression [55]. As depicted in Figure 5B, two distributions with relatively high residue importance are generally overlapped with DNA binding regions. Simultaneously, High-Mobility Group (HMG) domain, a compact DNA binding domain consists of 3 *α*-helice, has also been detected with GNNexplainer. cGMP-dependent 3′,5′-cyclic phosphodiesterase [56] and Serine/threonine-protein kinase B-raf [57] are two essential genes selected from *M. musculus* and the HepG2 cell line*.* Both are enzymes and have abundant functional sites such as initiator methionine, binding sites, active sites, post-transcriptional residues, etc. Figure 5C and D illustrates the distribution and overlapping situation of annotated key functional sites. As for cGMP-dependent 3′,5′-cyclic phosphodiesterase, the residues with relatively higher importance values are well mapped with those functional sites (Figure 5C). cGMP-specific phosphodiesterase, adenylyl cyclase, FhlA (GAF) and HDc domains have also been detected by finding/searching the connected subgraph with maximum connectivity with GNNExplainer. However, the functional sites of serine/threonine-protein kinase B-raf are not well characterized with the predicted distribution of residue importance, particularly for modification sites (cf. Figure 5D). Nonetheless, its functional domains, including Receptor Binding Domain (RBD) as well as C1, were identified during the post-training analysis employing GNNExplainer. These findings indicate that, even though Bingo was not designed or trained explicitly to identify key functional sites and/or structural domains, it has the potential to identify them via post-training analysis methods with the ultimate task of predicting essential genes.

## DISCUSSION

Herein, we have developed an LLM–GNN-based workflow, called Bingo, to predict gene essentiality of *C. elegans, D.*  *melanogaster*, *M. musculus* and *H. sapiens* (a HepG2 cell line) exclusively from protein sequence. Leveraging ESM-2 and GNNs, Bingo can harness complex and intrinsic patterns in protein sequences and contact maps, and achieves a high predictive performance based on comparative analyses, ablation studies and ‘zero-shot’ prediction. Importantly, Bingo can predict essential genes under a “zero-shot’ scenario using transfer learning, which means Bingo might be able to compensate for a lack of high-quality genomic and proteomic datasets for non-model organisms.

Notably, even without explicit annotations for amino acid residues, the present pipeline has the potential to infer intrinsic essentiality-linked elements, including functional sites and/or structural functional domains, via the attention mechanism and GNNExplainer, offering biological insights into the decision-making process of the model. Thus, Bingo should provide a promising tool for the prediction of essential genes within and among species, enabling the identification of novel intervention (drug or vaccine) candidates in socio-economically important parasites.

Nonetheless, Bingo might benefit from a more rigorous data pre-processing procedure and hypergraph model. Housekeeping genes [58], owing to their high conservation and widespread presence, may introduce bias, particularly in the cross-species essential gene prediction tasks. In future work, we aim to address this by collecting and using expanded experimental datasets, excluding highly conserved house-keeping genes, and focusing on species-specific genes during the data pre-processing phase. Furthermore, protein structures are complex and involve multiple levels of hierarchy, including primary, secondary, tertiary and quaternary structures. However, GNNs extract structure-level embeddings from a contact map and only provide a compressed two-dimensional map of a protein’s tertiary structure, which likely loses relevant information. Recently, hypergraph neural networks have emerged for enhanced protein structure and molecular interaction predictions [59–61]. These networks will likely be able to extract relevant multimodal structure information by allowing hyperedges to connect different types of nodes, capturing diverse relationships within the protein structures more efficiently than GNN models. These are areas that we plan to tackle in due course.

**Key Points**

- Bingo is an LLM–GNN-based workflow for the prediction of essential genes from protein datasets (both balanced and imbalanced) representing *C. elegans*, *D. melanogaster*, *M. musculus* and *H. sapiens* (i.e. a HepG2 cell line).
- This workflow uses ESM-2 to capture the contextual information from amino acid residues by considering their location (both upstream and downstream) in protein sequences, employs GAT to capture spatial correlations among residues in protein structures and is complemented by adversarial training.
- Bingo has a high performance for ‘zero-shot’ prediction, with *C. elegans* data being most reliable for training to learn and prioritize essential genes in an ‘unseen’ species.
- Bingo also exhibits excellent generalization and robustness when compared with three SOTA methods (DeepCellEss, EP-EDL and EP-GBDT) for cross-domain prediction using *C. elegans* data.
- Even without explicit annotations for proteins, Bingo has the potential to infer intrinsic essentiality-linked functional sites and structural functional domains via the attention mechanism and GNNExplainer, offering biological insight into the decision-making process.

## Supplementary Material

Bingo_Supplementary_File_BIB-23-1895_R1-FINAL_bbad472

## Data Availability

Processed protein data for *C. elegans*, *D. melanogaster*, *M. musculus* and the *H. sapiens* HepG2 cell line can be found at https://github.com/jianiM/Bingo. Python scripts for data processing, Bingo, comparative experiments and ablation studies are archived in https://github.com/jianiM/Bingo.

## Author Biographies

**Jiani Ma** is a joint PhD candidate in the Gasser Lab at the University of Melbourne and the School of Information and Control Engineering, China University of Mining and Technology. Her research interests include bioinformatics, machine learning and pattern recognition.

**Jiangning Song** (PhD) is an associate professor and group leader in the Monash Biomedicine Discovery Institute, Monash University, Australia. He is also affiliated with the Monash Data Futures Institute at Monash University. His research interests include bioinformatics, computational biomedicine, digital health, machine learning and pattern recognition.

**Neil D. Young** (PhD) is an associate professor and principal research fellow in the University of Melbourne. His research interests include the sequencing of genomes of cancer-causing parasites to understand the molecular biology of these pathogens and disease processes as well as developing enhanced bioinformatic tools for the annotation of genomes and gene sets.

**Bill C.H. Chang** (PhD) is a bioinformatician and the founder of the genetics company Yourgene Health. He is also an honorary senior research fellow at the University of Melbourne.

**Pasi K. Korhonen** (PhD) began as a software engineer at Nokia, and then pursued bioinformatics, earning his doctoral degree at the University of Melbourne. He is a senior research fellow and investigates parasites using genomic, transcriptomic and proteomic datasets.

**Tulio L. Campos** (PhD) has extensive experience with large-scale data analysis, focusing on systems engineering, machine-learning and genomics of prokaryotic and eukaryotic pathogens.

**Hui Liu** (PhD) is an associate professor in the School of Information and Control Engineering, China University of Mining and Technology. His research interests include bioinformatics, machine learning, artificial intelligence and software and hardware design of application systems.

**Robin B. Gasser** (PhD DVSc) is a fellow of the Australian Academy of Science (FAA) and a Redmond Barry distinguished professor at the University of Melbourne. His research focuses on understanding the biology of parasites, their interactions with their hosts and the diseases they cause using advanced molecular and informatic technologies.
