# Supplementary material for: ‘Bingo’—a large language model- and graph neural network-based workflow for the prediction of essential genes from protein data
Source: Brief Bioinform. 2023 Dec 27;25(1):bbad472. doi: 10.1093/bib/bbad472 (PMC10753293; doi:10.1093/bib/bbad472)
Supplement: Bingo_Supplementary_File_BIB-23-1895_R1-FINAL_bbad472 [file bingo_supplementary_file_bib-23-1895_r1-final_bbad472.docx]

# **‘*Bingo*’ - a large language model- and graph neural network (LLM-GNN)-based workflow for the prediction of essential genes from protein data**

**Supplementary Material**

Jiani Ma^1,2^, Jiangning Song^1,3^, Neil D. Young^1^, Bill C. H. Chang^1^, Pasi K. Korhonen^1^, Tulio L. Campos^1,4^, Hui Liu^2^, Robin B. Gasser^1,*^

^1^ *Department of Veterinary Biosciences, Melbourne Veterinary School, The University of Melbourne, Parkville, Victoria 3010, Australia*

^2^ *School of Information and Control Engineering, China University of Mining and Technology, Xuzhou, 221116, China*

^3^ *Monash Biomedicine Discovery Institute and Department of Biochemistry and Molecular Biology, Monash University, Melbourne, Victoria 3800, Australia*

^4^ *Bioinformatics Core Facility, Instituto Aggeu Magalhaes, Fundaçao Oswaldo Cruz (IAM-Fiocruz), Recife, Pernambuco, Brazil*

^*^To whom correspondence should be addressed: Robin B. Gasser. Email: robinbg@unimelb.edu.au

1. **The message passing mechanisms of GCN, GAT, GraphSAGE and GIN.**

**(i) GCN**

GCNs are spectral-based GNN models that work as a general form of CNN, extending grid-like images to arbitrary structural graphs. Here, we used GCN (cf. [1]), which simplifies previous GCNs by operating 1-localized convolution, and offers a compact and effective approach for graph-based feature extraction. For each protein, the message propagation rule was defined as:

$H^{(l+1)}=\sigma(\hat{D}^{-\frac{1}{2}}\hat{A}\hat{D}^{-\frac{1}{2}}H^{(l)}W^{(l)})$

where $\hat{A}=A+I$ is the protein contact map with added self-loops, while $\hat{D}$is the diagonal degree matrix of $\hat{A}$, is the residue-level embedding features in the *l*-th layer, and $H^{(0)}=X$. is a learnable parameter matrix. $\hat{D}^{-\frac{1}{2}}\hat{A}\hat{D}^{-\frac{1}{2}}$ is the process of neighbours’ message aggregation and normalization for each node. $\sigma(\hat{D}^{-\frac{1}{2}}\hat{A}\hat{D}^{-\frac{1}{2}}H^{(l)}W^{(l)})$ is a message passing and updating process. From the node-wise perspective, the node embedding rule can be defined as

$$h_{i}^{(l+1)}=\sigma(\sum_{j\in N(i)} \frac{1}{\sqrt{|N(i)|}\sqrt{|N(j)|}}W^{(l)}h_{j}^{(l)})$$

where ***N*(*i*)** is the set of one-localized neighbours of node *s*_i_, $\frac{1}{\sqrt{|N(i)|}\sqrt{|N(j)|}}$ is the normalization constant.

**(ii) GAT**

GAT regards some neighbours as more crucial than the others, rather than considering all neighbours as equally important [2]. Thus, rather than explicitly leveraging the statistically normalized convolution operation, GAT applies the attention mechanism to assign a different importance to each edge through the coefficients. Here, we utilized the GAT model proposed by Veličković [2]. The node-wise aggregation and updating rule is:$h_{i}^{(l+1)}=\sigma(\sum_{j\in N(i)} \alpha_{ij}^{(l)}W^{(l)}h_{j}^{(l)}),\text{ and }\alpha_{ij}^{(l)}=\frac{\exp(\mathrm{LeakyReLU}(a^{(l)^{T}}(W^{(l)}h_{i}^{(l)}||W^{(l)}h_{j}^{(l)})))}{\sum_{k\in N(i)} \exp(\mathrm{LeakyReLU}(a^{(l)^{T}}(W^{(l)}h_{i}^{(l)}||W^{(l)}h_{k}^{(l)})))}$

where $\alpha_{ij}^{(l)}$is the normalized attention coefficient which determines the relative importance of neighbouring features in relation to each other. is a learnable matrix; || denotes the concatenation operation; and is a learnable vector. In practice, the attention mechanism is a feed-forward neural network which is parameterized by $a^{(l)}$, and is activated by the LeakyReLU function. To explore versatile features, we leveraged GAT with two attention heads where the features generated by each head are concatenated.

$$h_{i}^{(l+1)}=\prod_{k=1}^{2} \sigma(\sum_{j\in N(i)} (\alpha_{k}^{(l)})_{ij}W_{k}^{(l)}h_{j}^{(l)})$$

**(iii) GraphSAGE**

Rather than considering all neighbours, GraphSAGE initially samples a fixed-size neighbourhood around the target node *i*. Subsequently, GraphSAGE updates the node *i*’s feature embeddings by concatenating the aggregated neighbourhood embedding and its current representation by performing a direct sum operation [3]. The updated formula for GraphSAGE is represented by:

$$\begin{matrix} h_{N(i)}^{(l+1)}=MEAN\{h_{j}^{(l+1)},\forall j\in S_{N(i)}\} \\ h_{i}^{(l+1)}=\sigma(W^{(l+1)}[h_{i}^{(l)}\oplus h_{N(i)}^{(l+1)}]) \end{matrix}$$

where MEAN denotes the mean aggregation function; ***S_N_*(*_i_*)** is the sampled immediate neighbours of node *i I*; $h_{N(i)}^{(l+1)}$is node *i*’s aggregated neighbourhood vector; $h_{i}^{(l)}$is the node *i*'s current representation; is the direct sum operator; and ***W***(*^l^*^+1)^ is a trainable parameter matrix.

**(iv) GIN**

GIN is a newly-proposed GNN model that shows major potential to capture graph structures [4]. Specifically, GIN uses the property of graph isomorphism *via* the protein contact map, and provides a graph at each GIN layer in the message-passing procedure. GIN is distinctly different from the three aforementioned GNN models in that it captures the graph structure while being invariant to node feature representations. Rooted in the isomorphic concept of the graph, GIN captures the node features by summing the aggregated neighbourhood representations, along with its own current representation. Then, for each protein, the resultant aggregated graph representation is passed through a fully connected linear network, typically with a non-linear activation function, to capture complex patterns and dependencies in the graph. The feature updating formula of GIN is:

$$h_{i}^{(k+1)}=MLP((1+\varepsilon^{(k+1)})h_{i}^{(k)}+\sum_{j\in N(i)} h_{j}^{(k)})$$

where $\varepsilon^{(k+1)}$ is a learnable scalar to extend the capacity of the trained model. ***MLP*** denotes a fully linear, connected neural network.

Different proteins have different numbers of nodes, which does not conform to batch computation. To mitigate this issue, we conducted the global max pooling operation after three GCN layers, to adjust and unify the size of graph embedding features. Finally, three consecutive, fully-connected layers were applied to the output of the global max pooling layer, while the first two were activated *via* the ReLU function; the final layer generated the output.

1. **Natural Language processing (NLP) benchmark methods utilised for comparative purposes**

For performance comparisons, we employed three representative NLP methods for benchmark models: Transformer [5], BiLSTM [6] and CNN[7]. These methods ensure the homogeneity of protein input data and, thus, allow for comparative analyses. The rationale for using these methods is provided in the following:

**Transformer**

Within the framework of sequence-to-sequence tasks, transformer has an encoder-decoder architecture [5]. Characterized by its stratified deployment of self-attention and feed-forward neural networks, the encoder captures long-range dependencies, thereby engendering contextualized representations of each constituent of the input sequence. The decoder in the transformer is responsible for generating output sequences based on contextual representations from the encoder. Because the prediction of essential genes is a binary classification task rather than sequence-to-sequence, we selectively utilised the encoder of the transformer to explore the contextual information embedded within individual protein sequences. Followed by the feed-forward neural network, using an ReLU activation function, transformer generated the final decision as to whether a gene is essential or not.

**BiLSTM**

BiLSTM [6] processes protein sequences by using specialised gating mechanisms to selectively retain and update information in both directions of each sequence simultaneously, allowing it to capture comprehensive information from amino acid residues. A fully-connected neural network was applied to a multi-layer BiLSTM to predict an essential protein-encoding gene.

**CNN**

Compared with the preceding two approaches, CNN is the most commonly used method for extracting contextual features from molecules, including protein sequences [8, 9]. Here, CNN was used to identify local patterns and features by using convolutional layers which apply filters to protein sequences and pools layers to down-sample feature representations, followed by a feed-forward neural network within CNN framework to predict a genes’ essentiality.

1. **Metrics used to assess model performance**

ROC and PR curves were utilized to intuitively evaluate the overall performance of *Bingo* and other existing methods. ROC curve shows the trade-off between *true positive rate* (*TPR*) and *false positive rate* (*FPR*), as the classification threshold varies, indicating how well the classifier can distinguish between positive and negative instances for various threshold settings [10]. The PR curve is more informative than AUROC when testing imbalanced data sets, highlighting the balance between precision and recall [11]. Area under the ROC curve (AUC) and area under the PR curve (AUPR), whose values range from 0 to 1, are computed based on the ROC and PR curves, respectively, providing a quantitative measurement of the overall performance of classifiers. Additionally, we also selected five commonly-used classification metrics, including the *F1 score*, *Accuracy* (*ACC*), *Recall*, *Specificity* and *Precision*, to assess the capability of *Bingo* and its competing models:

$$ACC=\frac{TP+TN}{TP+FN+TN+FP}$$

$$Precision=\frac{TP}{TP+FP}$$

$$Recall=\frac{TP}{TP+FN}$$

$$Specificity=\frac{TN}{TN+FP}$$

$$F1 score =\frac{2}{\frac{1}{Precision}+\frac{1}{Recall}}$$

where *TP*, *FP*, *TN* and *FN* denote the numbers of the true positive samples, false positive samples, true negative samples, and false negative samples, respectively.

Specifically, *ACC* calculates the ratio of correctly classified instances (both true positives and true negatives) to the total number of instances, providing an overall evaluation of correctness. *Recall* emphasizes the positive class, and measures the proportion of correctly identified positive instances out of all actual positive instances, whereas *Specificity* represents the proportion of correctly predicted negative instances out of all actual negative instances. *Precision* provides an assessment of the classifier's accuracy when it predicts an instance as positive, focusing on the misclassification of negative instances as positive. *F1 score* is a harmonic mean of *Precision* and *Recall*, also reflected in AUPR.

1. **Predictive performance using imbalanced data sets**

Using all annotated essential and non-essential genes (**Table 1**), we compared the performance of *Bingo* with the Transformer, BiLSTM and CNN pipelines by performing a stratified 10-fold cross-validation. The imbalanced dataset *scenario* aligns better with a practical situation, where the number of essential genes in an organism is usually smaller than that of non-essential genes. We provided the overall comparison results between *Bingo* and the three other methods using the four imbalanced datasets (representing *Caenorhabditis elegans*, *Drosophila melanogaster*, *Mus musculus* and HepG2 cell) with a stratified 10-fold cross-validation.

**[Fig. S1]**

As **Fig. S1A- S1D** show, in terms of AUROC, *Bingo* surpasses the three other methods by an average of 12.3%, 14.5%, 16.5%, 23.3% for *C. elegans*, for *D. melanogaster*, *M. musculus* and HepG2 cells, respectively. In terms of AUPR, when compared with BiLSTM (*C. elegans* and *D. melanogaster*), Transformer (*Mus musculus* and HepG2 cells), *Bingo* performed up to 30.6% better. Additionally, besides *Recall*, its performance was consistently high for the *F1 score*, *ACC*, *Precision* and *Specificity* (see **Fig. S1E**). When working in this imbalanced data setting, where the number of non-essential genes is greater than that of the essential genes, proceeding without sampling non-essential genes and pre-existing false-negative samples resulted in a distinct performance degradation. Nonetheless, *Bingo* had the best performance and reliability for the prediction of essential genes compared with the three other methods.

1. **State-of-the-art (SOTA) methods for cross-domain experiment using *C. elegans* protein data**

*Bingo* takes protein sequence data as input. To ensure a fair and rigorous comparison, we selected three SOTA methods that were given protein sequences. These methods, namely DeepCellEss [12], EP-EDL[13] and EP-GBDT [14], were chosen for a comprehensive evaluation in the context of cross-domain prediction using data representing the model organism *C. elegans*.

Specifically, we employed the balanced *C. elegans* dataset derived from OGEE database as the training set (i.e., source domain). To create an independent test dataset (i.e., the target domain), we extracted essential genes from the Database of Essential Genes (DEG)[15], which contains records of 338 essential genes of *C. elegans*. As non-essential genes were not available in the DEG database, we randomly sampled 338 non-essential genes from a published study [16]. This combined balanced dataset, comprising the DEG essential genes and the sampled non-essential genes, constituted our new test dataset, referred to as the “DEG-Ravi” test set. The latter test set was smaller than the training set. After training and validating *Bingo*, along with other SOTA models, on the training and validation sets, we proceeded to assess the models’ predictive capabilities using DEG-Ravi. Their brief introduction and the details about how we implemented them in cross-domain experiment re described in the following:

**DeepCellEss**

DeepCellEss [12] is an attention-based interpretable deep learning framework, designed for cell line-specific essential gene prediction. It accepts protein sequence data as input; sequence data are transformed into one-hot encoding matrices. Subsequently, a CNN module is employed to capture local features in the sequences. Then, to enhance the interpretability, a multi-head self-attention module is utilized to generate residue-level attention scores, locating the most important sequence regions that contribute to essentiality *versus* non-essentiality. Furthermore, the incorporation of dual skip-connections, enveloping the CNN and multi-head self-attention, is developed to mitigate potential model degradation concerns. Thereafter, a BiLSTM module is engaged to model sequential data, facilitating the acquisition of long-range dependencies. Finally, the prediction of essential genes is executed after max-pooling, followed by a fully connected layer.

Although it is specifically designed for cell lines, the model architecture of DeepCellEss fits the task of essential gene prediction of any species. Here, we extended its use to *C. elegans* data. During the training procedure, protein sequences were observed to vary in from length. To standardize the input for the batch operation, we set 800 amino acids as the cutting-off threshold. For protein sequences longer than 800 amino acids, we truncated them at the length of 800 aa, while for protein sequences that were shorter than 800 amino acids, we padded them with a padding tag. Taking the standardised *C. elegans* protein sequence data as input for DeepCellEss, we re-run and optimized the system using the training and validation sets which were split from balanced *C. elegans* dataset from OGEE. Finally, we test the optimized DeepCellEss on the assembled DEG-Ravi test set.

**EP-EDL**

EP-EDL is a CNN-rooted ensemble deep learning model, which is designed for the prediction of essential genes in humans. EP-EDL takes the Position-Specific Scoring Matrix (PSSM) features extracted from protein sequences as input, and employs the multi-scale convolutional layers with varying kernels to capture higher-level features. The resultant multi-scale features are aggregated and then processed *via* a fully connected linear neural network for final predictions.

In the context of the cross-domain experiment using *C. elegans* data, we implemented EP-EDL by generating PSSM [17] matrices for each protein in the balanced *C. elegans* dataset from OGEE and the assembled DEG-Ravi test set using the PSI-Blast command line [18] . A PSSM matrix represents the probability of finding a specific amino acid at each position within a sequence. Specifically, each row represents a position within the sequence, and 20 natural amino acids are 20 columns in PSSM matrix.[17]. Due to variations in protein length, PSSM sizes differ. To facilitate batch processing, we standardized PSSM sizes by capping protein sequences at 800 amino acids. For protein sequences that were longer than 800 amino acids, we retained only the first 800 rows, while for those with a length of less than 800 amino acids, we padded the remaining rows with zeros. As a result, each protein sequence is represented by its own PSSM matrix with 800 rows and 20 columns, enabling efficient batch processing. In the training procedure, the original EP-EDL model employed an ensemble learning strategy to mitigate prediction bias due to imbalanced datasets. In this experiment, given the use of balanced datasets, we refrained from employing the ensemble learning strategy recommended in the original research for both model training and testing.

**EP-GBDT**

EP-GBDT [14] is a machine learning ensemble model for the prediction of essential huma genes . It begins by extracting Pseudo amino acid composition (PseAAC) features from protein sequences. These features serve as input for EP-GBDT, which employs an ensemble learning strategy consisting of multiple Gradient Boosting Decision Tree (GBDT) classifiers. In this experiment, we did not implement the ensemble training strategy, as explained previously.

In the context of our cross-domain experiment using *C. elegans* data, we reconfigured EP-GBDT as follows: we initially extracted PseAAC features from all protein sequences using iFeature [19] software. Subsequently, we applied EP-GBDT with a single GBDT classifier to the training set. We employed a grid search strategy to identify the optimal combination of hyperparameters, which resulted in an *n_estimators* being set at 100, *learning_rate* at 1.2, and *max_depth* at 5. This optimized EP-GBDT configuration was then deployed on the DEG-Ravi test set.

To assess the predictive capability of our cross-domain prediction, we adopted a Top-K gene scheme. In this scheme, we ranked the predicted probability scores for all genes in the test set in a descending order. For each method, we then recorded the cumulative, correctly predicted essential genes within the top 10%, top 20%, top 30%, top 40%, and top 50% of the ranked genes. **Fig. S2** visualizes the performance generated by *Bingo*, DeepCellEss, EP-EDL and EP-GBDT.

[**Fig. S2**]

As **Fig. S2** shows, across all ratios, *Bingo* consistently outperformed the other methods by correctly identifying more essential genes. DeepCellEss followed as the second-best performer, maintaining a significant lead over EP-EDL and EP-GBDT. Additionally, when focusing on the top 50% of ranked genes, *Bingo* identified 294 essential genes, accounting for 86% of all essential genes in test set (338 in total in the DEG-Ravi test set). These findings indicate the effectiveness of *Bingo* for prioritizing essential genes.

**References**

1. Kipf TN, Welling M. Semi-supervised classification with graph convolutional networks, In: 5th International Conference on Learning Representations(ICLR), Toulon, France, 2017.

2. Veličković P, Cucurull G, Casanova A et al. Graph Attention Networks, 6th International Conference on Learning Representations(ICLR), Vancouver, Canada, 2018.

3. William L. Hamilton, RY., Jure Leskovec. Inductive Representation Learning on Large Graphs, in Proceedings of the 31st International Conference on Neural Information Processing Systems(NeurIPS), Long Beach, UA USA, 2018.

4. Keyulu Xu WH, Jure L, Stefanie J. How powerful are graph neural networks? arXiv:1810.00826, 2019.

5. Vaswani A, Shazeer N, Parmar N et al. Attention Is All You Need, In: 31st Annual Conference on Neural Information Processing Systems (NeurIPS), Long Beach, USA, 2017.

6. Shu Z, Dequan Z, Xinchen H et al. Bidirectional Long Short-Term Memory Networks for Relation Classification. In: Proceedings of the 29th Pacific Asia Conference on Language, Information and Computation, 2015. pp. 73–78, Shanghai, China.

7. Kim Y. Convolutional Neural Networks for Sentence Classification, Proceedings of the 2014 Conference on Empirical Methods in Natural Language Processing (EMNLP). Association for Computational Linguistics, Doha, Qatar, 2014. pp. 1746-1751.

8. Tsubaki M, Tomii K, Sese J. Compound-protein interaction prediction with end-to-end learning of neural networks for graphs and sequences. Bioinformatics 2019; 35: 309-318.

9. Van den Broeck L, Bhosale DK, Song K et al. Functional annotation of proteins for signaling network inference in non-model species. Nat Commun. 2023; 14: 4654.

10. Fawcett T.An introduction to ROC analysis. Pattern Recognition Letters 2006; 27: 861-874.

11. Saito T, Rehmsmeier M. The Precision-Recall Plot Is More Informative than the ROC Plot When Evaluating Binary Classifiers on Imbalanced Datasets. PloS One 2015;10: e0118432.

12. Li YM, Zeng M, Zhang FH et al. DeepCellEss: cell line-specific essential protein prediction with attention-based interpretable deep learning, Bioinformatics 2023;39: btac779.

13. Li YM, Zeng M, Wu YF et al. Accurate Prediction of Human Essential Proteins Using Ensemble Deep Learning, Ieee-Acm Transactions on Computational Biology and Bioinformatics 2022;19:3263-71.

14. Zeng MaW, Nian and Wu, Yifan and Li, Yiming and Wu, Fang-Xiang and Li, Min. Improving human essential protein prediction using only protein sequences via ensemble learning, IEEE International Conference on Bioinformatics and Biomedicine (BIBM) 2021:98-103.

15. Luo H, Lin Y, Liu T et al. DEG 15, an update of the Database of Essential Genes that includes built-in analysis tools, Nucleic Acids Research 2021;49:D677-D86.

16. Kamath RS, Fraser AG, Dong Y et al. Systematic functional analysis of the Caenorhabditis elegans genome using RNAi, Nature 2003;421:231-37.

17. Henikoff JG, Henikoff S. Using substitution probabilities to improve position-specific scoring matrices, Computer Applications in the Biosciences 1996;12:135-143.

18. Altschul SF, Madden TL, Schaffer AA et al. Gapped BLAST and PSI-BLAST: a new generation of protein database search programs, Nucleic Acids Research 1997;25:3389-402.

19. Chen Z, Zhao P, Li FY et al. iFeature: a Python package and web server for features extraction and selection from protein and peptide sequences, Bioinformatics 2018;34:2499-502.

**Figure Legends**

**Fig. S1. Comparison of the overall performance of *Bingo* and other methods using imbalanced data (10-fold cross-validation).** **A-D**. The ROC and PR curves of *Bingo* (and its competing methods on balanced datasets for *C. elegans*, *D. melanogaster*, *Mus. musculus* and *Homo sapiens* HepG2 cell line. **E**. Performances of *Bingo*, Transformer, BiLSTM and CNN using four balanced data sets, in terms of *ACC*, *Precision*, *Recall*, *F1 score* and *Specificit*y (10-fold cross-validation).

**Fig. S2.** **Performance comparison results employing the Top-K gene scheme.**
